# Identification and Antimicrobial Susceptibility of *Mycoplasma capricolum* subsp. *capripneumoniae* Isolates from China During 2024–2025

**DOI:** 10.3390/vetsci13030229

**Published:** 2026-02-27

**Authors:** Zilong Cheng, Leilei Yang, Yanna Wei, Wenwen Zhang, Yuzi Wu, Maojun Liu, Fusheng Si, Chunhua Li, Zhixin Feng, Wenliang Li

**Affiliations:** 1 Key Laboratory of Veterinary Biological Engineering and Technology, Ministry of Agriculture and Rural Affairs, Institute of Veterinary Medicine, Jiangsu Academy of Agricultural Sciences, Nanjing 210014, China; 20210005@jaas.ac.cn (Z.C.); 20120034@jaas.ac.cn (L.Y.); 20100021@jaas.ac.cn (Y.W.); 20110978@jaas.ac.cn (W.Z.); 20110982@jaas.ac.cn (Y.W.); 20030022@jaas.ac.cn (M.L.); 2 GuoTai (Taizhou) Center of Technology Innovation for Veterinary Biologicals, Taizhou 225300, China; 3 Institute of Animal Husbandry and Veterinary Science, Shanghai Academy of Agricultural Sciences, Shanghai 201106, China; mr.fusheng@163.com (F.S.); lichunhua@saas.sh.cn (C.L.)

**Keywords:** *Mycoplasma capricolum* subsp. *capripneumoniae* (Mccp), *Mycoplasma ovipneumonia* (Mo), phylogenetic analysis, MLSA, antimicrobial susceptibility

## Abstract

Respiratory diseases induced by *Mycoplasma* species, mainly *Mycoplasma capricolum* subsp. *capripneumoniae* (Mccp) and *Mycoplasma ovipneumonia* (Mo), pose a major threat to goat/sheep farming. This study investigated the biological characteristics and antimicrobial susceptibility of Mccp isolates that suddenly spread extensively in China in the first half of 2024. A total of 34 Mccp isolates were obtained from goats with suspected contagious caprine pleuropneumonia (CCPP) across multiple provinces during 2024–2025. All isolates were purified and confirmed via a PCR targeting the *arcD* gene and exhibited characteristic “fried egg” colony morphology. Phylogenetic analysis based on the *arcD* gene and multi-locus sequence analysis (MLSA) of eight genetic loci revealed that the circulating strains shared high homology and belonged to Group 1 within Lineage 1, which showed a close genetic relationship with isolates from Qatar and the United Arab Emirates, while differing from previously reported strains in China. Antimicrobial susceptibility testing against nine antimicrobial drugs indicated that the Mccp isolates generally exhibited low resistance levels. However, some strains showed reduced susceptibility to florfenicol and lincomycin. These findings highlight the emergence of a genetically distinct Mccp lineage in China and underscore the importance of ongoing surveillance, strain characterization, and prudent antimicrobial use in CCPP control.

## Simple Summary

This study characterized *Mycoplasma capricolum* subsp. *capripneumoniae* (Mccp), the pathogen that causes contagious caprine pleuropneumonia (CCPP), in goats across China during 2024–2025. A total of 34 Mccp strains were isolated and analyzed. Genetic analysis showed that these strains are different from previous Chinese strains and are closely related to isolates from Qatar and the United Arab Emirates. These isolates generally showed low resistance to most antimicrobials, with several strains presenting resistance to florfenicol and lincomycin. These findings suggest a new type of Mccp is spreading in China, highlighting the need for continued monitoring and rational use of vaccines and drugs in goat farming.

## 1. Introduction

China is a major sheep/goat-raising country, with both the stock number and the slaughtered number ranking first in the world [1]. With the increasingly intensive and large-scale development of the goat/sheep industry in China, respiratory diseases occur frequently and are relatively complex. A wide range of pathogens contribute to the clinical respiratory diseases, including Mo, caprine parainfluenza virus type 3 (CPIV3), mannheimia haemolytica, Pasteurella, and Mccp. Compared to other animal diseases, research on the epidemiological features, etiological characteristics, and pathogenesis of respiratory diseases in goat/sheep was relatively scarce.

Mccp is the causative agent of CCPP, a severe and economically devastating, highly contagious respiratory disease in goats, characterized by high morbidity/mortality in affected goat flocks, endemic in Africa, the Middle East, and Asia. CCPP is a specific acute disease in goats, characterized by fever, cough, fibrinous pneumonia, and pleurisy lesions, and is a notifiable disease under the World Organisation for Animal Health (WOAH) [2]. CCPP was first described by Thomas in 1873, and its contagious nature was demonstrated by Hutcheon in 1881. In 1976, MacOwan successfully isolated Mccp for the first time in Kenya, designated it as F38, and successfully reproduced CCPP in goats, naming the pathogen Mccp, accordingly [3].

In 1935, China reported the outbreak of contagious pleuropneumonia in goats [4]. Subsequently, the disease spread across various provinces, including Inner Mongolia, Ningxia, Gansu, Tibet, Sichuan, Shandong, Zhejiang, Liaoning, and Fujian. From 1949 to 1989, more than 890,000 goats contracted the disease, and over 320,000 succumbed to it [5]. In the 1950s, the lungs of Mccp-positive goats from Xinjiang, Shandong, and the Shanxi province of China were collected, and the Mccp isolates were subcultured in goats or chicken embryos for the production of a vaccine. The prevalence of Mccp in China has decreased and become sporadic since then [4,5,6,7,8]. At the beginning of 2024, there were lots of cases of goats with respiratory diseases/sudden death cases and the clinical and pathological features were consistent with those of CCPP (data from Bureau of Animal Husbandry and Veterinary Services of the Ministry of Agriculture and Rural Affairs of China). The causative pathogen was subsequently identified as Mccp.

Mccp belongs to the class Mollicutes and the *Mycoplasma mycoides* cluster and is a fastidious bacterium for isolation. Due to the lack of effective isolation media and techniques, the isolation of Mccp is always difficult and unsuccessful from naturally infected goats in CCPP endemic countries. So, there is relatively little research regarding the prevalence, etiological and genomic characteristics, and pathogenesis of Mccp worldwide. In addition, the drug resistance of Mo and *Mycoplasma mycoides* subsp. *capri* (Mmc) was reported [9,10,11], but there has been no report performed on Mccp.

In the present study, pathological examination and Mccp isolation and identification were performed for the suspected CCPP cases during 2024–2025. The purified isolates were subjected to biological characterization, genetic evolutionary analysis, and antimicrobial resistance testing.

## 2. Material and Methods

### 2.1. Clinical Samples and Reagents

Clinical samples were collected from goats suspected of having CCPP from 19 goat farms in Jiangsu, Anhui, Shandong, Zhejiang, Hebei, Henan, Shanghai, and Xinjiang. The goats have not been vaccinated against CCPP or were vaccinated at least half a year ago. The dead or moribund goats exhibited symptoms such as loss of appetite, lethargy, panting, and difficulty breathing. After the necropsy of the deceased goats, the lungs and/or pleural effusion were collected and kept at 4 °C until transportation to the laboratory. The collected samples were immediately examined by PCR, the positive PCR samples were subjected to mycoplasma isolation, while the others were stored at −80 °C. The KM2 cell-free liquid medium, a modified Friis medium, contains 15% (*v*/*v*) swine serum, 0.5% pyruvate (*w*/*v*), and 500 U/mL of penicillin, supplemented with 0.005% phenol red (*w*/*v*) as a growth indicator, was prepared by our laboratory and used for Mccp isolation. The KM2–agar medium was prepared by adding 1% agar (Biowest Agarose^®^G-10; Gene Company Limited, Chi Wan, Hong Kong) to KM2 liquid medium and used for Mccp purification. Nine antimicrobials from different classes (enrofloxacin, ciprofloxacin, doxycycline, florfenicol, tiamulin, tylosin, tilmicosin, tylvalosin, and lincomycin) were purchased from MDBio, Inc. (Qingdao, China). The drugs were diluted to stock solutions according to the Clinical and Laboratory Standards Institute criteria in their specific solvents for antimicrobial sensitivity testing.

### 2.2. Pathological Analysis

The dead goats were dissected, and the lesions in various tissues and organs were observed and recorded. Lung samples showing typical CCPP lesions were collected for histological investigations. All tissue samples were placed immediately in 10% neutral buffered formalin for 72 h for routine histological examination. Samples were embedded in paraffin wax, sectioned at 4 μm, and stained with hematoxylin and eosin. Only animals that have been dead for no more than 24 h have been sampled to ensure the best quality of the sample. Microscopic examination was performed using the Pannoramic MIDI II digital slide scanning system (3DHISTECH, Budapest, Hungary).

### 2.3. DNA Extraction and PCR-Based Detection

Samples from lung lesions and/or pleural effusions and isolated strains were collected for molecular tests. Genomic DNA was extracted using the Bacteria Genomic DNA Kit (Catalog No. CW0552S, CWBio, Inc., Beijing, China) according to the manufacturer’s instructions and then subjected to a PCR test. Primers used in the study included Spe forward (5′-ATCATTTTTAATCCCTTCAAG-3′) and Spe reverse (5′-TACTATGAGTAATTATAATATATGCAA-3′), which were utilized to amplify a 316 bp fragment of the Mccp *arcD* gene, as previously described in references [12,13]. Additionally, primers for the Mo *16S rRNA* gene were employed: 16S forward (5′-GAAGTCTTTCGGGATGTA-3′) and 16S reverse (5′-TTTGTAGTAGCCATTGTAGC-3′), targeting an 826 bp fragment of the desired gene. For the *Mannheimia haemolytica lktD* gene, primers Mh forward (5′-GCAGGAGGTGATTATTAAAGTGG-3′) and Mh reverse (5′-CAGCAGTTATTGTCATACCTGAAC-3′) were used to amplify a 206 bp fragment, following previously described methods [14]. Lastly, primers Pm forward (5′-ATCCGCTATTTACCCAGTGG-3′) and Pm reverse (5′-GCTGTAAACGAACTCGCCAC-3′) were applied to amplify a 456 bp fragment of the *Pasteurella multocida KMT1* gene, consistent with earlier protocols [15]. The 2× Rapid Taq Master Mix was used for PCR, and thermal cycling was performed according to the manufacturer’s instructions (Vazyme, Nanjing, China): an initial denaturation step of 95 °C for 3 min, followed by 35 cycles at 95 °C for 15 s, 47 °C (for Mccp) or 50 °C (for Mo) for 15 s, and 72 °C for 15 s, with a final elongation step at 72 °C for 10 min. Amplification patterns were analyzed using UV transilluminator.

### 2.4. Mccp Isolation and Identification

Lungs and pleural effusions samples that tested positive for Mccp by PCR were further used for Mccp isolation. Lung samples (1–2 g) were ground in 2 mL PBS and centrifuged at 4000× *g* for 5 min. The supernatants or pleural effusions were filtered through a 0.45-micron filter. Approximately 0.2 mL of the filtrates was inoculated into 2 mL KM2 liquid medium and incubated at 37 °C, having 5% CO_2_ for 5–10 days. The medium color change was checked every day. Once the color changed from red to yellow, the inoculations were diluted and inoculated on the KM2–agar medium for Mccp purification. The KM2–agar medium was checked daily until visible single colonies appeared on the plate. Then, a single colony was picked into the KM2 liquid medium for proliferation. The purification process was repeated to obtain purified Mccp strains. The isolates were propagated in KM2 liquid medium and identified by PCR as described in 2.3. The titers were tested for color change unit (CCU) as previously described [16]. Each isolate was divided into aliquots and frozen at −80 °C.

### 2.5. Sequencing and Phylogenetic Analysis

To determine the genetic relationship of the Mccp isolates, the *arcD* gene fragments amplified by PCR were sequenced, and the phylogenetic analysis was performed using MEGA12 (version 12.1.2). The phylogenetic tree was constructed using the maximum likelihood method with 1000 bootstrap replications. To enhance the understanding of the epidemiology of epidemic Mccp strains in China, whole-genome sequencing was conducted on three representative Mccp isolates from different provinces in China by Novogene (Beijing, China). Multi-locus sequence analysis (MLSA) based on the analysis of eight genetic markers was performed as previously reported [17,18]. The locus sequences corresponding to each strain were concatenated head-to-tail for diversity analyses conducted using Darwin 6.0 [17,19]. A distance tree was constructed using the neighbor-joining algorithm. Early Chinese Mccp strains 87001 and M1601 and foreign representative strains were included in the analysis.

### 2.6. Antimicrobial Susceptibility Testing

Nine antibiotics from different classes were tested: enrofloxacin (fluoroquinolones), ciprofloxacin (fluoroquinolones), doxycycline (tetracyclines), florfenicol (amphenicols), and tiamulin (pleuromutilin); and members of the macrolides: tylosin, tilmicosin, tylvalosin, and lincomycin (lincosamides). Antimicrobial susceptibility was evaluated by the minimal inhibitory concentration (MIC) test using a broth microdilution assay as previously described [20]. Briefly, the stock drug solutions were diluted to the initial working concentration: 32 μg/mL (tylosin, doxycycline, tiamulin, enrofloxacin, ciprofloxacin, and tylvalosin), 64 μg/mL (florfenicol and lincomycin), and 128 μg/mL (tilmicosin). Then, the drugs were diluted by a 2-fold serial dilution to 12 gradients and added to columns 1 to 12 of a 96-well polystyrene microtiter plate (100 μL/well). The Mccp isolates grown to the logarithmic phase were diluted to 10^4^ ccu/mL and added (100 μL/well) to the plate containing drugs. Each isolate was tested in three replicate wells for each drug. Control wells of Mccp growth (only Mccp), drug control (only drug), and KM2 medium control were set up. Subsequently, the plates were sealed with a plate-sealing film and incubated for 7 days. When the Mccp growth control well turns yellow, and the drug control well and KM2 medium control well do not change color, the test is valid. The lowest drug concentration corresponding to the wells that remain unchanged in color was the MIC value. The mean MIC values were obtained through three independent repeated experiments. Mccp 87001 strain was used as a quality control strain to monitor the performance of the MIC test.

## 3. Results

### 3.1. Clinical Signs and Autopsy Findings in CCPP-Suspected Goats

**Figure 1:**
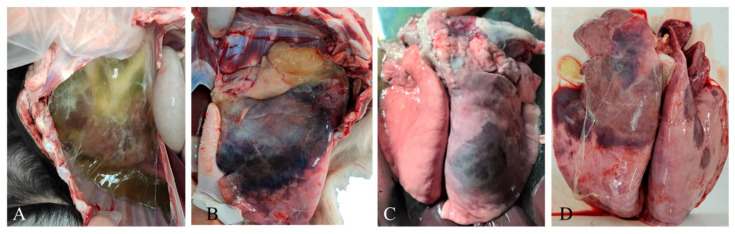
Autopsy lesions of the clinically dead goats. (**A**) A large amount of gelatinous fluid accumulation in the chest cavity; (**B**–**D**) enlarged and firm lungs with fibrinous pleuropneumonia; and bilateral or unilateral cranio-ventral pulmonary dark red consolidation and a large amount of fibrinous exudate covering the surface of the lungs.

Based on the inquiry and investigation of the goat farmers, the incidence rate of the disease in affected goat farms was 30–90%, and the mortality rate was 5–60%. The sick goats showed symptoms of fever, depression, anorexia, and respiratory signs such as nasal discharges, occasional cough, dyspnea and polypnea, especially after strenuous activity. If no treatment measures are taken, most of the infected goats begin to die within a few days after the onset of the aforementioned symptoms. Sometimes, the goats may suddenly die without showing any obvious clinical symptoms. The post-mortem examination revealed varying degrees of lung and pleural lesions. Severely affected goats presented with a significant accumulation of light yellow pleural effusion within the thoracic cavity, accompanied by bilateral or unilateral dark red pulmonary consolidation (Figure 1A,B,D). The pleural fluid rapidly gelatinized upon exposure to air (Figure 1A). The affected lung was enlarged, firm, and edematous (Figure 1A,B,D), and cross-sectioning the affected lung reveals a widened interlobular septa and exudative edema within the interlobular spaces. On cut surfaces, lobular areas appeared to be “granular”. The goats with milder disease presented only with the unilateral consolidation of the cranio-ventral lobes of the lung (Figure 1C). The surface of some of the lungs was covered with a layer of fibrinous exudate (Figure 1B,D) and manifested as various degrees of fibrinous pleuropneumonia. In severe CCPP cases, pericardial effusion may be observed. All samples tested positive for MCCP and negative for *Pasteurella multocida*. Three samples tested positive for *Mannheimia haemolytica*, and two tested positive for Mo.

### 3.2. Histopathological Observation

**Figure 2:**
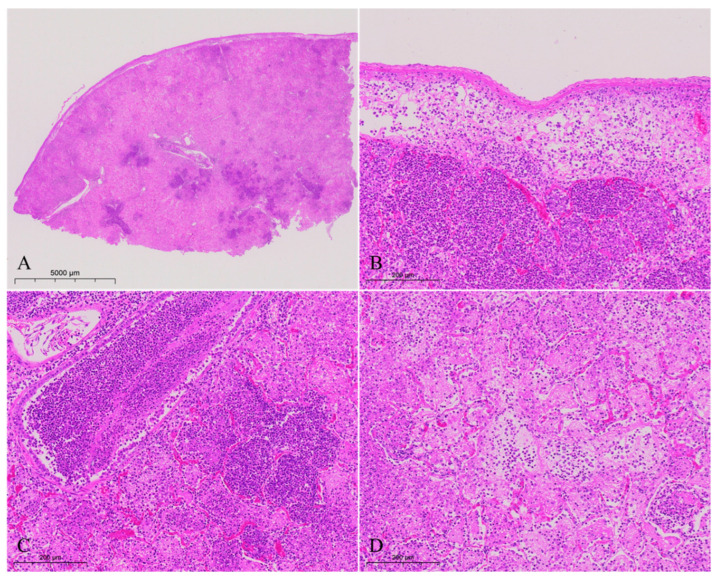
The histopathological findings in the lungs of diseased goats. (**A**) Diffuse consolidation of the entire section with loss of normal architecture. (**B**) Hyperplasia and thickening of the pulmonary serosa, overlaid with fibrinous exudate; inflammatory cells infiltration and fibrin exudation under the serous membrane and in the lumen of the alveoli of the lung; and capillary hyperemia in the alveolar walls. (**C**) Capillary hyperemia in the alveolar walls; a lot of neutrophils, along with small numbers of macrophages, shed epithelial cells, and cellular debris in the lumen of the bronchus and lumen of alveoli. (**D**) Lots of fibrinous exudation and inflammatory cells in the lumen of alveoli.

The lungs exhibited severe lesions characterized by diffuse consolidation and a loss of normal architecture throughout the entire section (Figure 2A). The pulmonary serosa showed hyperplasia and thickening, overlaid with fibrinous exudate. Beneath the serosa, numerous inflammatory cells including macrophages and neutrophils were present, along with fibrinous exudate and cellular debris (Figure 2B). Capillaries within the alveolar walls showed hyperemia, and alveolar cavities and bronchial lumens were filled with neutrophils, along with small numbers of macrophages, shed epithelial cells, and cellular debris (Figure 2B–D). Alveolar cavities were also filled with abundant fibrinous exudate (Figure 2D). The walls of some alveoli and bronchioles were necrotic, with epithelial cells undergoing necrosis and shedding into the lumen of the alveoli (Figure 2B,D) or bronchioles (Figure 2C).

### 3.3. Identification of Mccp Isolates

**Figure 3:**
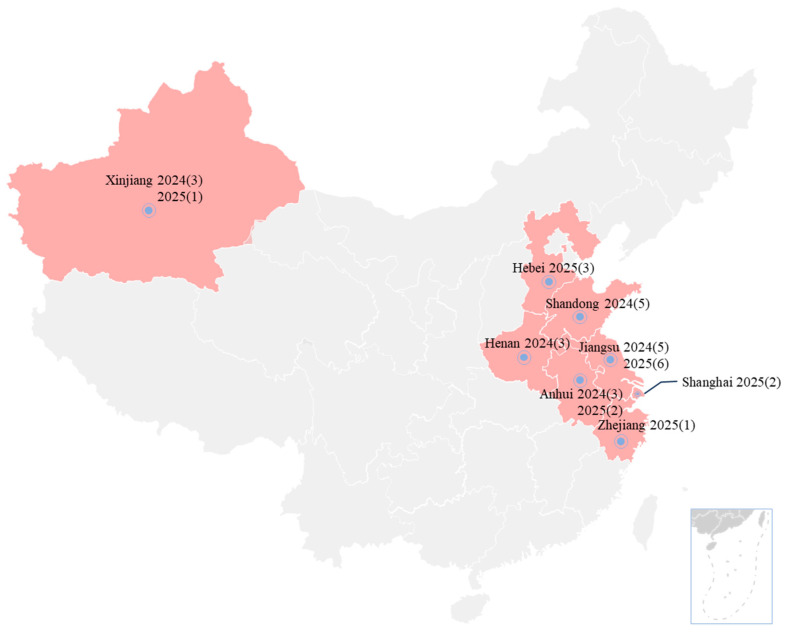
Distribution of the Mccp isolates in this study. The numbers in the brackets indicate the Mccp isolate number of the specific province and year.

**Figure 4:**
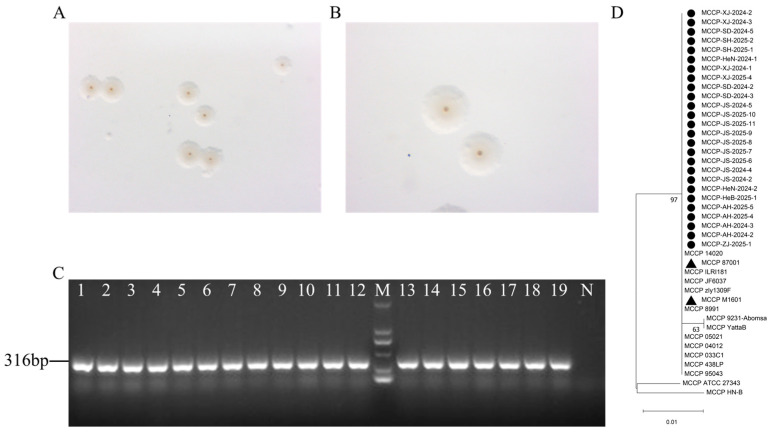
Isolation and identification of the Mccp isolates and genetic analysis with reference strains. (**A**,**B**) Typical fried egg-like colonies with a small central core spot of the Mccp isolates; (**A**) 20×; and (**B**) 40×. (**C**) PCR identification of the Mccp isolates. M: DNA DL2000 marker; N: negative control; and Lanes 1–19: Mccp isolates. (**D**) Genetic analysis of the Mccp isolates from China and reference strains based on the *arcD* gene. The black dot indicates the strains isolated here. The black triangle indicates the previously reported Chinese strains (87001 and M1601).

One to three Mccp-positive samples from each goat farm were selected for Mccp isolation. From May 2024 to December 2025, thirty-four Mccp isolates were successfully isolated from 19 goat farms in ten different provinces (Figure 3). No Mo strain was isolated. Detailed information on the Mccp isolates is indicated in Supplemental Table S1. The purified Mccp isolates showed typical fried egg-like, large granular colonies with a small central core spot (Figure 4A,B). After 1–2 passages, all of the isolates showed a medium color change from red to yellow within 5 days. All isolates were confirmed as Mccp by a specific PCR with an amplicon of 316 bp (Figure 4C). These PCR amplification products were sequenced and subjected to NCBI Blast. All of them were identified as Mccp and shared 100% homology. Phylogenetic analysis of these sequences along with the reference sequences of domestic and foreign strains revealed high homology among them, clustering into a single branch (Figure 4D).

### 3.4. Multi-Locus Sequence Analysis of Mccp Isolates

**Figure 5:**
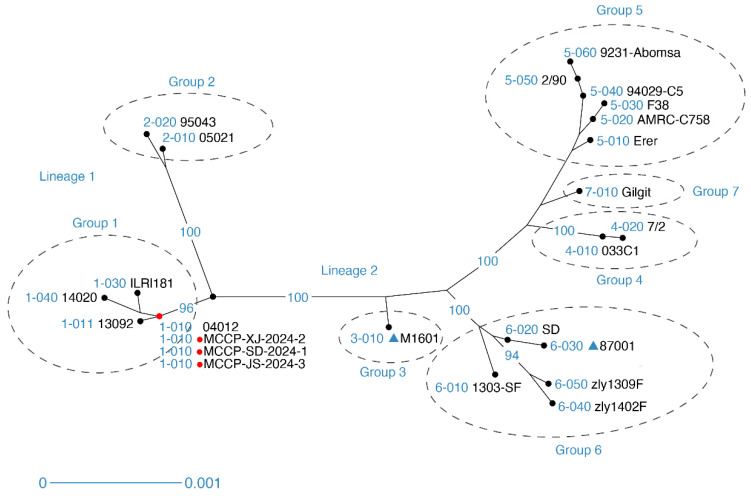
A phylogenetic tree created based on a distance analysis of the eight concatenated MLSA loci. Eight MLSA loci corresponding to each strain were concatenated head-to-tail for diversity analyses conducted using Darwin 6.0. A distance tree was constructed using the neighbor-joining algorithm. The red dot indicates the strains isolated in this study. The previously reported Chinese strains (87001 and M1601) are labeled with a blue triangle.

As the phylogenetic analysis of the *arcD* gene sequences did not effectively distinguish inter-strain differences, MLSA based on whole-genome sequencing was conducted. Three selected isolates from different provinces were sequenced and used for MLSA, together with the 21 reference Mccp strains (Supplemental Table S2). A robust tree (Figure 5) was obtained from the distance analysis of MLSA data using DARwin6. The tree inferred from the concatenated sequences of the eight selected loci revealed that the analyzed strains can be divided into two lineages consisting of seven groups. Lineage 1 included Groups 1 and 2; Lineage 2 included Groups 3 to 7. Unexpectedly, all three isolates belonged to Group 1 of Lineage 1, which has primarily been associated with epidemics in Qatar, the United Arab Emirates, and parts of East Africa. Notably, these isolates did not belong to Group 6 or Group 3, which had previously been epidemic in China. This indicated that the currently circulating strains were significantly different from those in the past in China. Furthermore, the concatenated sequences of the eight selected loci from these three Mccp isolates, sourced from different provinces and time periods, exhibited 100% homology. The origins and virulence of these newly isolated Mccp strains require further research to be elucidated.

### 3.5. MICs of Mccp Isolates

**Figure 6:**
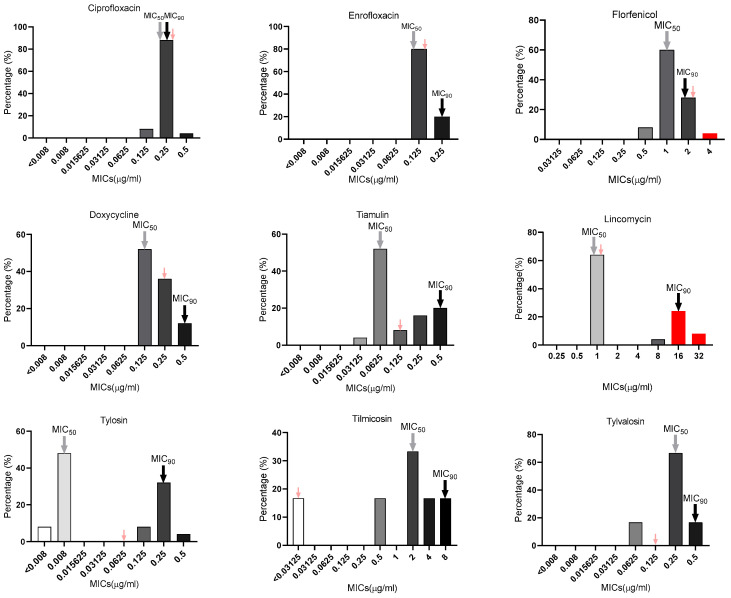
The MICs distribution of nine antimicrobials to Mccp isolates and reference strain 87001. The X-axis indicates concentration of the antimicrobials; the Y-axis indicates the percentage of isolates in the specific antimicrobial concentration. The gray and black arrows above the histogram indicate the MIC_50_ and MIC_90_, respectively. The pink arrow above the histogram indicates the MIC of the Mccp reference strain 87001. The red bars represent drug-resistant strains.

Based on antimicrobial susceptibility test results, the distribution of MIC, MIC_50_, and MIC_90_ values was calculated and presented in Figure 6. The MIC values for each drug against every MCCP strain are provided in Supplemental Table S3. The MIC values for the reference strain 87001 were 0.25 μg/mL for ciprofloxacin and doxycycline; 0.125 μg/mL for enrofloxacin, tiamulin, and tylvalosin; 2 μg/mL for florfenicol; 1 μg/mL for lincomycin; 0.0625 μg/mL for tylosin; and <0.03125 μg/mL for tilmicosin. The MIC range of the nine antimicrobials for these Mccp isolates were 0.125–0.5 μg/mL (ciprofloxacin), 0.125–0.25 μg/mL (enrofloxacin), 0.5–4 μg/mL (florfenicol), 0.125–0.5 μg/mL (doxycycline), 0.03125–0.5 μg/mL (tiamulin), 1–32 μg/mL (lincomycin), <0.008–0.5 μg/mL (tylosin), 0.03125–8 μg/mL (tilmicosin), and 0.0625–0.5 μg/mL (tylvalosin). According to the standard of the Pasteurellaceae intermediate clinical breakpoints for cattle of florfenicol (≥4 μg/mL) and lincomycin (≥16 μg/mL) [9,10], some Mccp isolates were antimicrobial-resistant strains. The isolates did not show resistance to other tested drugs. Lincomycin and tilmicosin had a relatively high MIC_50_ (1 μg/mL for lincomycin and 2 μg/mL for tilmicosin) and MIC_90_ (16 μg/mL for lincomycin and 8 μg/mL for tilmicosin). Overall, apart from lincomycin, the drug resistance of the prevalent Mccp isolates was not significant.

## 4. Discussion

CCPP has been commonly detected in domestic and wild small ruminant animals across Asia, Africa, and Europe, especially in Asia and Africa, since it was first identified [21]. Compared to the hazards of pneumonia caused by Mo, CCPP has a more severe effect on the goats. Mo can cause chronic bronchointerstitial pneumonia both in sheep and goats [22]. Since Mo is a common microorganism in the respiratory tract of sheep and goats, the morbidity rate is usually high, but the mortality rate is low [23]. Compared to the mild clinical symptoms and pathological findings caused by Mo, Mccp can lead to severe fatal hemorrhagic fibrinous pneumonia and pleurisy. The morbidity and mortality rate associated with Mccp could reach as high as 100% [21,24]. In the last several decades, with the high coverage of vaccination, CCPP has sporadically occurred in China. In 2024, CCPP began to emerge as outbreaks and frequently occurred afterwards (data from Bureau of Animal Husbandry and Veterinary Services of the Ministry of Agriculture and Rural Affairs of China). This study aims to investigate the molecular and biological characteristics of the strains associated with the epidemic.

Mccp is a member of the *Mycoplasma mycoides* cluster, which includes several species, subspecies, and strains: Mccp, *Mycoplasma capricolum* subsp. *capricolum* (Mcc), *Mycoplasma mycoides* subsp. *capri* (Mmc), *Mycoplasma mycoides* subsp. *mycoides* type SC (MmmSC), and *Mycoplasma leachii* (M. leachii). MmmSC and M. leachii are associated with bovine diseases, while Mccp, Mcc, and Mmc are linked to caprine diseases. Both Mccp and MmmSC can cause infectious pleuropneumonia. In contrast, Mmc, Mcc, and M. leachii can lead to a syndrome known as ‘MAKePS syndrome’, which stands for mastitis, arthritis, keratitis, pneumonia, and septicemia. In the laboratory, Mccp can be easily confused with the closely related capricolum subspecies. In 1988, Chinese researchers used the Mmc model strain PG3, MmmSC model strain PG1, *Mycoplasma agalactiae* model strain PG2, and Mo model strain Y98 as controls to identify the Chinese isolates from CCPP-like cases in the1950s by using growth inhibition assays and indirect fluorescence methods. They found that the isolates had the closest relationship with PG3 and therefore classified the pathogen of CCPP in China as Mmc [25]. Until 2007, these isolates were re-identified as Mccp through molecular biology methods [5]. With the widespread application of molecular biology techniques, the test and identification of Mccp is faster and easier. The presence of Mccp in scattered CCPP cases has been reported in China [8,26,27,28], even though, due to the difficulty in culturing, research on the etiological characteristics and pathogenesis of Chinese Mccp strains is still limited. In this study, by using the modified KM2 medium prepared in our lab, Mccp was isolated from the positive PCR samples with a >95% success rate. These isolates showed a rapid growth rate and high titers and exhibited the typical colony morphology of Mccp. The medium could be a good choice for Mccp isolation.

Phylogenetic analysis of *arcD* gene fragments revealed 100% homology within the prevalent isolates. By utilizing the concatenated sequences of the eight selected loci analysis method established by Manso-Silván [18], all Mccp strains can be divided into two lineages and seven groups, where Lineage 1 includes Groups 1 and 2 and Lineage 2 includes Groups 3 to 7 (Figure 5). Moreover, the MLSA group of Mccp strains is closely related to their geographic distribution [17,18]. The eight concatenated MLSA loci analysis results are largely consistent with the findings from whole-genome analysis [29,30], indicating that this method serves as a good tool for analyzing the genetic evolution of Mccp. Three selected isolates identified in this study all belong to Group 1 within Lineage 1, which is different from the previously reported Group 3 and Group 6, which are prevalent in China [17,18]. Prior to this, Group 1 epidemic strains were predominantly circulating in Qatar and the United Arab Emirates in Western Asia, as well as in certain areas of East Africa [17,31,32]. In addition, the prevalent strain in China is closely related to the 04012 strain from Qatar, both belonging to the 1-010 types in the MLSA typing system (Supplemental Table S2, Figure 5). Building upon these findings, comprehensive genomic epidemiological investigations should be prioritized to clarify the origin and evolutionary trajectory of the 1-010 type strains currently circulating in China. Whole-genome sequencing combined with phylogeographic analysis would enable the higher-resolution tracing of transmission routes and provide insights into possible cross-border dissemination events. In particular, comparative genomic analyses between Chinese isolates and strains reported in Western Asia and East Africa may help determine whether recent outbreaks resulted from independent introductions or long-term undetected circulation. The 04012 strain from Qatar and the 13092 strain from the United Arab Emirates were both isolated from wild animals. Additionally, cases of wild Tibetan antelopes dying due to CCPP were reported in China in 2012 [33]. This inevitably raises the possibility that the novel MCCP strains currently spreading widely in China after 2024 may have been introduced through wildlife. In addition to molecular surveillance, strengthened monitoring of wildlife populations and cross-species transmission interfaces is essential. The potential role of wild ungulates as reservoirs or bridge hosts warrants systematic investigation, especially in regions where domestic goats and wild ruminants share grazing areas. Long-term ecological surveillance programs could help identify risk factors associated with spillover events and clarify whether wildlife plays a primary or secondary role in maintaining Mccp transmission cycles. Due to the lack of prevalence data and information on Mccp in Central Asian countries, the real cause of the CCPP epidemic in China after 2024 requires further investigation. By conducting MLSA on new Mccp isolates in China, we have further uncovered the unforeseen diversity of Mccp in Asia and the likelihood of its prevalence within the continent. Further research is required to explore the relationship between the currently prevalent 1-010-type strains and their virulence, along with their transmission rate. Such research is of critical safety significance for investigating the prevalence and routes of Mccp’s transboundary transmission, and it also holds substantial global importance for the prevention and control of Mccp. Moreover, unlike the results obtained from the phylogenetic analysis of the *arcD* gene, the MLSA analysis revealed significant differences between the epidemic strains and the existing vaccine strains (87001, M1601) in China. However, the differences in antigenicity and pathogenicity have yet to be tested in future studies. Although our investigations over the past two years indicate that currently available commercial vaccines continue to provide a certain degree of clinical protection against CCPP, most clinical cases are associated with delayed vaccination or expired vaccine protection. Therefore, timely immunization and appropriate booster administration should be prioritized to ensure effective protection. Nevertheless, the exact protective efficacy of various commercial vaccines against emerging epidemic strains in China still requires further validation through well-designed and systematic animal experiments. Future research should focus on comprehensive antigenic characterization and cross-protection assessments between circulating field isolates and vaccine strains. These studies should include measurements of humoral and cellular immune responses, clinical symptom monitoring, pathogen load quantification, and pathological examinations to comprehensively assess protection levels. Detailed genomic and proteomic analyses of emerging epidemic strains will help identify potential mutations or antigenic variations that may affect vaccine-induced immunity. Such data will provide a scientific basis for evaluating whether current vaccine formulations require updating or optimization. Collectively, these efforts will contribute to a more comprehensive understanding of Mccp evolution and epidemiology in Asia and provide a scientific basis for the prevention and control of future CCPP outbreaks.

Currently, there are reports regarding drug resistance in various *Mycoplasma* species such as Mo, *Mycoplasma bovis*, Mmc, etc. [9,10,11,34,35,36,37], but no such reports have been performed concerning Mccp. On goat farms, in addition to vaccine immunization, the prevention and treatment of CCPP and other respiratory diseases primarily relies on the use of antimicrobials such as macrolides, tetracyclines, fluoroquinolones, amphenicols, and lincosamides. Therefore, it is crucial to determine the resistance of Mccp to commonly used antimicrobials to effectively guide the prevention and control of clinical diseases. According to results from this study, the currently prevalent strains of Mccp are sensitive to macrolides, tetracyclines, and fluoroquinolones. However, similar to Mo [9], some Mccp strains exhibit a certain resistance to florfenicol and lincomycin, but Mccp’s resistance to florfenicol is relatively weak, while its resistance to lincomycin is more pronounced. Resistance to tetracyclines and Macrolides–Lincosamides–Streptogramin–Ketolides (MLSK) in animal *Mycoplasma* species is mainly associated with point mutations in ribosomal targets [9]. But the determinative resistance mechanism of Mccp remains to be further studied. Although the isolated strains in this study were highly homologous, their MIC values already exhibited significant differences, suggesting varying rates of resistance development during transmission across different goat herds. It is anticipated that more resistant strains will emerge in the future. The use of sensitive drugs for prevention and early treatment can significantly reduce both the severity and mortality associated with the disease. Nevertheless, the potential for antimicrobial misuse contributing to resistance should not be overlooked. In many endemic regions, antimicrobials are frequently administered empirically, often without laboratory confirmation of the causative agent or antimicrobial susceptibility testing. Inappropriate drug selection, subtherapeutic dosing, shortened treatment courses, and the prophylactic overuse of antimicrobials can all create selective pressure that accelerates the emergence of resistant strains. Such practices compromise treatment efficacy and increase the risk of persistent infection, relapse, and further transmission within herds. To mitigate these risks, rational antimicrobial use must be emphasized. This includes promoting evidence-based treatment guided by laboratory diagnostics, implementing antimicrobial susceptibility testing when feasible, and adhering strictly to recommended dosages and treatment durations.

## 5. Conclusions

In conclusion, the prevalent strains of CCPP in China are distinct from those identified in the past. It is important to investigate the prevalence and transmission characteristics of these novel strains both in China and other Asian countries. Additionally, the infection of wild animals with Mccp should also be of concern. An eight-gene concatenated analysis offers a valuable method for studying the genetic evolution of novel Mccp strains. Currently, there are no severe drug-resistant strains of Mccp reported; however, varying degrees of drug resistance have been observed among other *Mycoplasma* species. Therefore, it is crucial to maintain vigilance regarding the emergence of drug resistance in Mccp, as well as to establish specific standards for drug resistance and clarify the mechanisms behind it.

## Data Availability

The original contributions presented in this study are included in the article/Supplementary Materials. Further inquiries can be directed to the corresponding authors.

## Supplementary Materials

The following supporting information can be downloaded at: https://www.mdpi.com/article/10.3390/vetsci13030229/s1, Table S1: The table of MCCP isolates information; Table S2: The table of reference MCCP strains and MCCP isolates information analyzed in this study and corresponding to MLSA types; Tables S3: The MIC values for each drug against every strain.
